# A revision of the *Megachile* subgenus *Litomegachile* Mitchell with an illustrated key and description of a new species (Hymenoptera, Megachilidae, Megachilini)

**DOI:** 10.3897/zookeys.221.3234

**Published:** 2012-09-13

**Authors:** Emily L. Bzdyk

**Affiliations:** 1 Bohart Museum of Entomology, University of California, Davis, California, 95616, USA

**Keywords:** Revision, Mexico, leafcutting, bee, *Megachile*, morphology, nomenclature, illustration, key

## Abstract

The species of *Megachile* subgenus *Litomegachile* are revised with a review of the species morphology, biology, and plant associations. A new species, *Megachile pankus*, is described and illustrated. *Megachile mendica snowi* Mitchell is elevated to species. *Megachile* var. *nupta* Cresson and *Megachile texana* var. *cleomis* Cockerell are synonymized with *Megachile brevis* and *Megachile texana*, respectively. An illustrated key for *Litomegachile* is also provided.

## Introduction

*Litomegachile* is a subgenus of *Megachile* Latreille, a large genus including leafcutting and resin bees. Leafcutting bees are solitary and get their name from their habit of using leaf pieces and other plant materials to form the lining of their nests (Michener 2007). Although sometimes difficult to separate from other *Megachile*, certain combinations of characters can be useful in identifying *Litomegachile*. For males, the combination of fore coxal spines present, mandible tridentate, forelegs slender, unmodified and a tomentum of white hair on the sixth metasomal tergum is diagnostic. In females the combination of mandible with face dull in apical half, four-toothed (sometimes with dorsal tooth subtruncate), with distinct cutting edge between 2^nd^ and 3^rd^ teeth, sixth sternum with apical margin not upturned, scopal hairs uniformly covering ventral surface, and metasomal sterna lacking apical fringes of white hair separates them from other *Megachile*.

**Figure 1.**
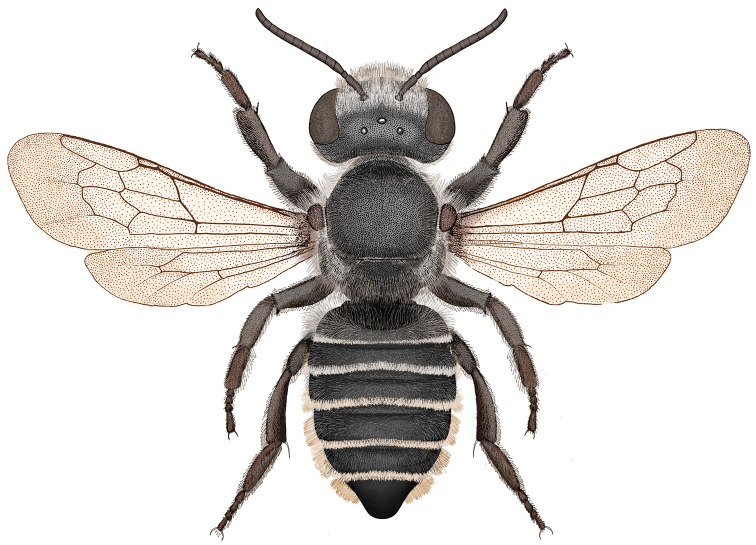
Illustration of *Megachile pankus* dorsal view.

**Figure 2.**
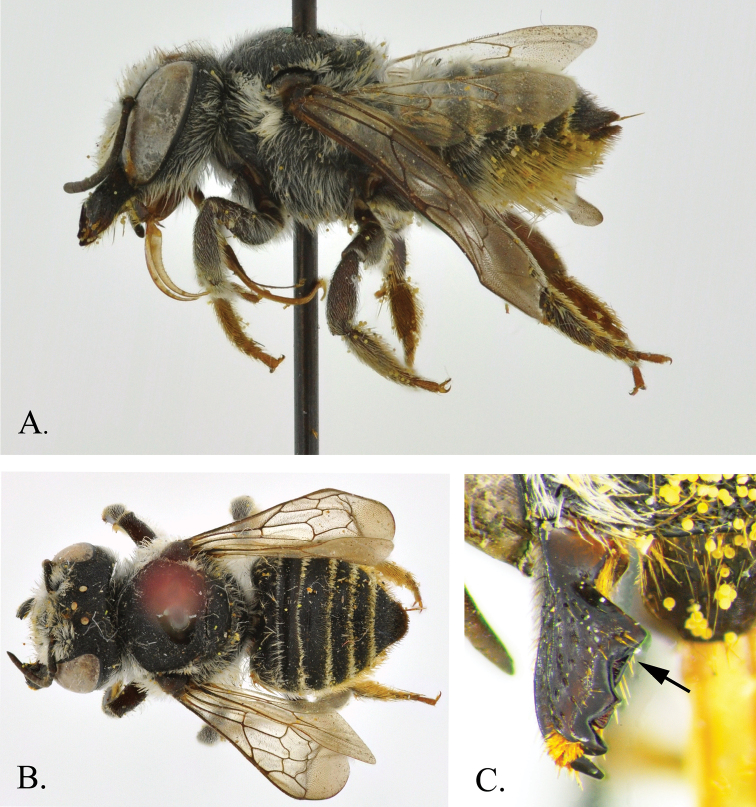
*Megachile pankus* **A** Lateral view **B** Dorsal view **C** Mandible showing angulation.

The subgenus was first described by Mitchell (1935). He provided a key to five species: *Megachile brevis* Say, 1837, *Megachile coquilletti* Cockerell, 1915, *Megachile gentilis* Cresson, 1872, *Megachile mendica* Cresson, 1878, and *Megachile texana* Cresson, 1878, and six infraspecific taxa: *Megachile mendica* var. *snowi* Mitchell, 1927, *Megachile brevis* var. *onobrychidis* Cockerell, 1908, *Megachile brevis* var. *nupta* Cresson, 1872, *Megachile brevis* var. *pseudobrevis* Mitchell, 1936, *Megachile texana* var. *cleomis* Cockerell, 1900,and *Megachile texana* var. *lippiae* Cockerell, 1900. There is a questionable record from Peru, that Mitchell named *Megachile buchwaldi* Mitchell, but it was never described and no type was ever designated (Raw 2004), so it is a *nomen nudum*. Sheffield et al. (2011) published a key to the *Megachile* of Canada in which he raised *Megachile onobrychidis*, *Megachile lippiae* and *Megachile pseudobrevis* to species level.Specimens from Mexico identified as *Megachile onobrychidis* and other unidentified specimens were found to be a new species, *Megachile pankus*, described below (Figures 1–2). Ten species are recognized here.

The life history and nesting biology of *Litomegachile* species is relatively well known (Michener 1953, Krombein 1967, Baker et al. 1985, Packer 1987). These bees are cavity nesters, usually choosing an existing cavity in wood, a plant stem, or the ground, where they construct a nest of several individual cells. The cells are arranged in a linear fashion, and are cylindrically shaped. Leaf or petal pieces are used to form a cup shape, and are often glued together by the female bee biting the edges to create adhesion (Krombein 1967). Kim (1992) found that cell size determines how much pollen is provisioned, which in turn determines the size of the resulting offspring. These bees follow the pattern shown in many other solitary bees which construct a linear nest. The females are larger than the males, and are placed in the rear of the nest behind the males, since they usually take longer to develop and emerge (Kim 1992).

*Litomegachile* has been considered a Nearctic subgenus (Mitchell 1935). However, at least *Megachile brevis*, *Megachile lippiae* and *Megachile pankus* have ranges that extend into the Neotropical Region. The local distribution of these bees may be strongly tied to favorable floral blooms, and may change throughout the season and from year to year (Michener 1953).

## Materials and methods

Type depository collections are given in the text as the following acronyms: AMNH - American Museum of Natural History, New York, New York, USA; ANSP - Academy of Natural Sciences, Philadelphia, Pennsylvania, USA; BBSL - Bee Biology and Systematics Lab, Logan Utah, USA; BMEC - Bohart Museum of Entomology, University of California, Davis, California, USA; BMNH -The Natural History Museum, London, United Kingdom; USNM - U.S. National Museum, Washington, D. C. USA; MCZ - Museum of Comparative Zoology, Harvard University, Cambridge, Massachusetts, USA; NCSU - North Carolina State University, Raleigh, North Carolina, USA; UCMC – University of Colorado Museum, Boulder, Colorado, USA. Approximately 1,300 Specimens were examined from the AMNH, BMEC, BBSL, and USNM. Primary type specimens were examined for *Megachile cleomis*, *Megachile coquilletti*, *Megachile cleomis var. lippiae*, *Megachile murinella*, *Megachile onobrychidis*, *Megachile pseudobrevis*, *Megachile schismatura*, and *Megachile snowi*. Type specimens of *Megachile pankus* sp. n. are deposited in BBSL, AMNH, and BMEC.

Distribution maps were created using data from Discover Life’s online mapping program (Ascher and Pickering 2011). Records were included from the collections of AMNH, BMEC, BBSL, and USNM, as well as those that were determined by experts from other collections. Other specimen records exist, including those from Kansas University, Berkeley, San Diego, Los Angeles, Riverside and Chamela. Due to funding and time limitations, determinations were not confirmed for many of the records from these collections and therefore were not included in these maps. The book “Biodiversidad, taxonomía y biogeografía de artrópodos de México” provides state level records of *Litomegachile* for Mexico that are not included here (Ayala et al. 1997).

**Figure 3.**
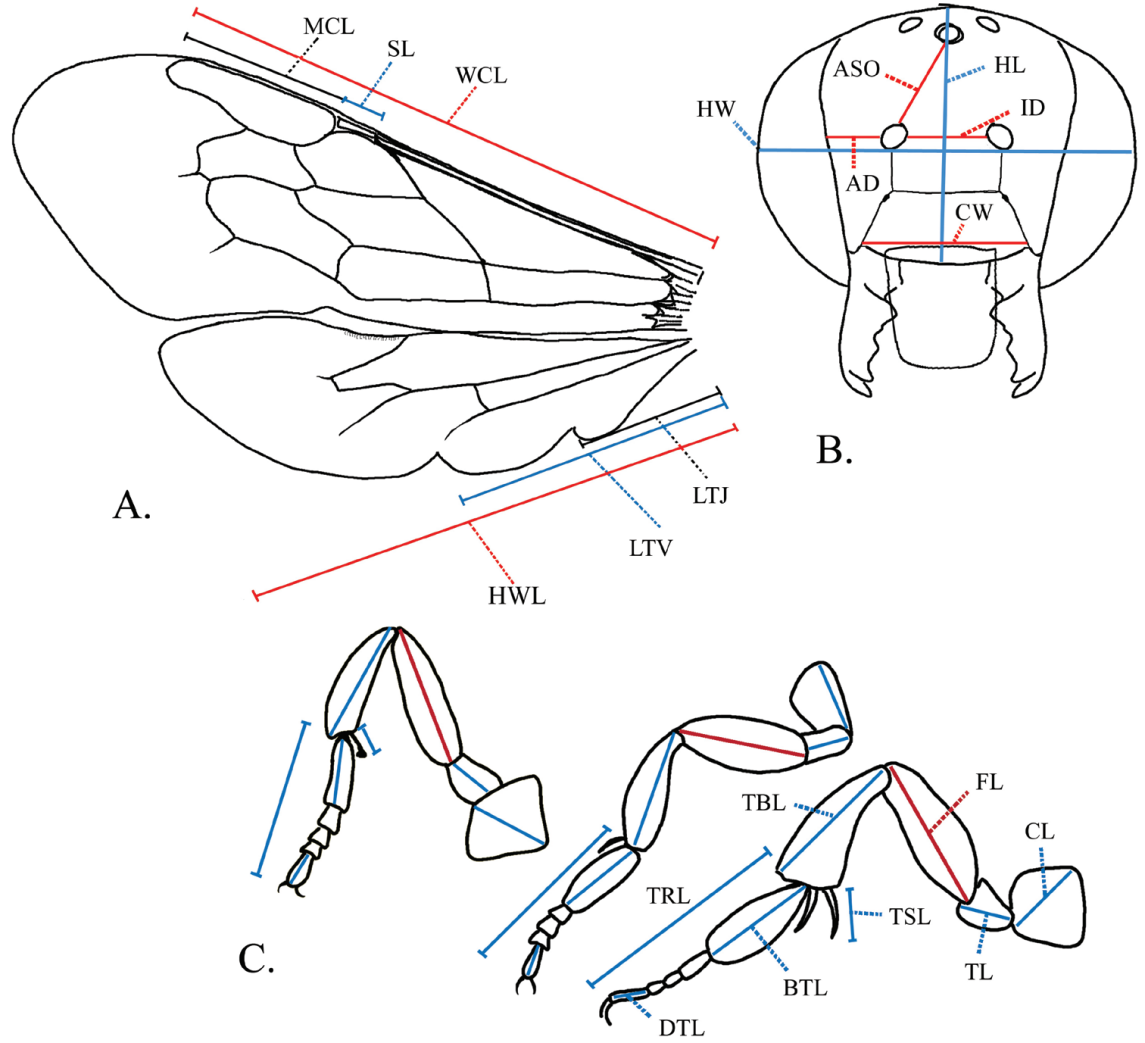
Measurement points for *Megachile pankus* **A** Wing measurements **B** Head measurements **C** Foreleg, midleg and hindleg measurements. Abbreviations: **MCL**=marginal cell length **SL**= stigma length **WCL**= wing cells length **HWL**=hind wing length **LTV**=length to vannal lobe **LTJ**= length to jugal lobe **HW**= head width **HL**=head length **ASO**= distance from antennal socket to anterior ocellus **AD**= antennocular distance **ID**= interantennal distance **CW**=clypeus width **DTL**=distitarsus length **TRL**=tarsus length **BTL**=basitarsus length **TSL**=tibial spur length **TBL**=tibia length **FL**=femur length **TL**=trochanter length **CL**=coxa length.

Plant classification and families for flower records follow that of the USDA Plants Database (http://plants.usda.gov/java/).Morphological terminology and measurements follows that of Michener (2007). Metasomal tergum 5 is given as “T5”, metasomal sternum 6 is given as “S6” and flagellomere 1 as “F1”. Head length is measured from the vertex to the apical clypeal margin. Head width is measured from the outer margins of compound eyes when viewed from the front (Figure 3B). Mandible teeth are numbered inward, with most distal tooth being number one. Ratios between width and length are given as a decimal for different dimensions of segments. Ratios of leg segment lengths are measured at the longest point, and compared to the respective femur length (Figure 3C). Antennocular distance is the width of the paraocular area from compound eye to the antennal socket. Interantennal distance is the width of the supraclypeal area between the antennal sockets (Figure 3B). The T6 transverse carina is a structure in males at the functional apex of the metasoma. It arises from the medial discal area of T6 and terminates in a notched or irregularly jagged edge (Figure 6C–I). Below the carina of T6, is the true apical margin, with four teeth: two submedial and two lateral (Figure 6A–B). The tomentum is a patch of white hair on T6 of males that is thick enough to hide the discal surface. Pubescence is defined as branched body hairs, such as those found on head, mesosoma, and discal surfaces of metasoma and apical fringes of hair of tergal segments. Setae are those unbranched, “eyelash-like” hairs found on the metasoma along the margins of tergal segments, and that make up the scopa on the sterna of females. Abbreviations used for measurements as illustrated in Fig. 3 are as follows: MCL=marginal cell length, SL= stigma length, WCL= forewing length in region with cells, HWL=hind wing length, LTV=length to vannal lobe, LTJ= length to jugal lobe, HW= head width, HL=head length, ASO= distance from antennal socket to anterior ocellus, AD= antennocular distance, ID= interantennal distance, CW=clypeus width, DTL=distitarsus length, TRL=tarsus length, BTL=basitarsus length, TSL=tibial spur length, TBL=tibia length, FL=femur length, TL=trochanter length, CL=coxa length.

**Figure 4.**
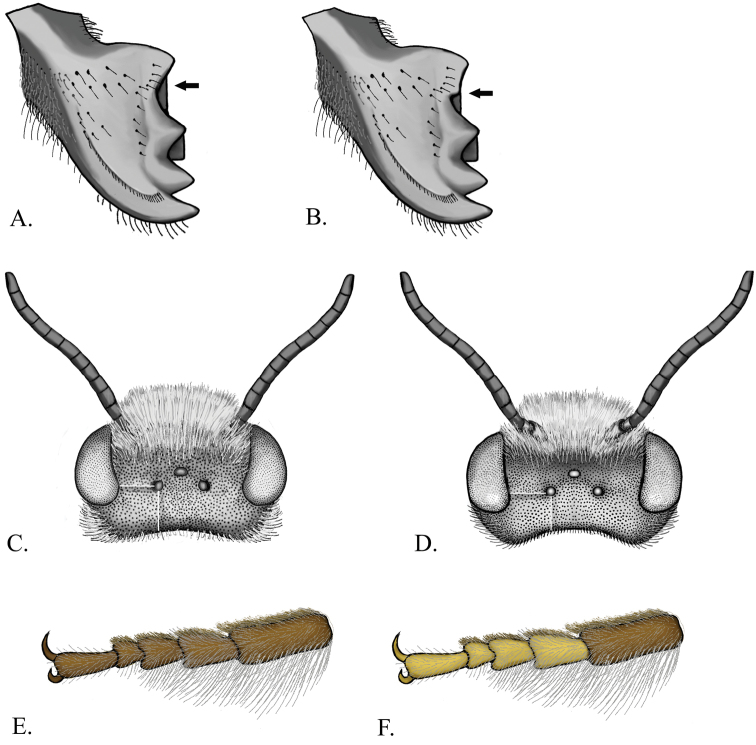
**A** Female mandible with even concavity **B** Female mandible with angulation **C** male *Megachile texana* head dorsal view of ocelli distances **D** Male *Megachile brevis* head dorsal view of ocelli distances **E** *Megachile brevis* front tarsus **F** *Megachile coquilletti* front tarsus

### Key to the species of *Litomegachile*

**Females**

**Table:** 

1	Mandible angulate between teeth 3 and 4 (Figure 4B)	2
–	Mandible evenly concave between teeth 3 and 4 (Figure 4A)	5
2(1)	S6 scopa entirely black; S5 scopa black apically, rest of scopa ivory (Figure 5C); metasomal terga with complete transverse apical fringes of white hairs	*Megachile gentilis* Cresson
–	S6 scopa partially black, rest of scopa yellow (Figure 5D, 5F, 5H); metasomal terga with partial apical fringes of white hairs.	3
3(2)	T6 concave laterally and in profile, with erect setae arising above appressed black pubescence toward base in profile (Figure 5F)	*Megachile pankus* sp. n.
–	T6 slightly concave laterally, straight in profile and without erect setae (Figures 5D, 5H)	4
4(3)	T6 with pale appressed pubescence (Figure 5H)	*Megachile snowi*Mitchell
–	T6 with brownish appressed pubescence (Figure 5D)	*Megachile mendica* Cresson
5(1)	T6 slightly concave laterally and in profile (Figure 5B)	*Megachile coquilletti* Cockerell
–	T6 strongly concave laterally and in profile	6
6(5)	T6 with evenly concave slope in profile, with white appressed hair, and black erect setae basally (Figure 5I)	7
–	T6 convex basally, then concave apically in profile; hair and setae variable (Figures 5A, 5E, 5G)	8
7(6)	Only T5-T6 with black setae on lateral margins in dorsal view (Figure 5J)	*Megachile lippiae* Cockerell
–	T2-T6 with black setae on lateral margins in dorsal view (Figure 5K)	*Megachile texana* Cresson
8(6)	S6 scopa mostly ivory, with few if any blacksetae apically; T6 with white appressed hair apically (Figure 5A)	*Megachile brevis* Say
–	S6 scopa black; T6 with black appressed pubescence	9
9(8)	S1-S5 scopa ivory; southwestern United States (Figure 5G)	*Megachile pseudobrevis* Mitchell
–	S5 bearing apical black hairs; Western United States and Canada (Figure 5E)	*Megachile onobrychidis* Cockerell

**Males**

**Table:** 

1	Apical margin of T6 (not transverse carina) with submedian teeth closer to each other than to lateral teeth, or distances equal (Figure 6A)	2
-	Apical margin of T6: submedian teeth closer to lateral teeth than each other (Figure 6B)	4
2(1)	Punctures on surface of T6 near the edges of tomentum crowded, edges form between depressions (Figure 6C); T2 with apical fringe of white hair (Figure 6J)	*Megachile gentilis* Cresson
–	Punctures on surface of T6 not crowded, shiny surface apparent between depressions (Figure 6D). T2 with no apical fringe of hair or fringe only present laterally (Figure 6K)	3
3(2)	T5 with complete apical fringe of white hair (Figure 6E)	*Megachile snowi* Mitchell
–	T5 without apical fringe of white hair (Figure 6D)	*Megachile mendica* Cresson
4(1)	Foretarsal segments 2-4 yellow, contrasting with dark basal segment (Figure 4F); T5 with incomplete apical fringe (Figure 6F)	*Megachile coquilletti* Cockerell
–	Entire front tarsi brown, tarsal segments not contrasting in color (Figure 4E); T5 with complete apical fringe	5
5(4)	Ocellocular distance equal to ocelloccipital distance (Figure 4D)	6
–	Ocellocular distance less than ocelloccipital distance (Figure 4C)	8
6(5)	T6 with a white tomentum that obscures the discal surface (Figure 6G)	7
–	T6 without tomentum, or if tomentum present, sparse, tergal surface visible beneath white hairs (Figure 6H)	*Megachile onobrychidis* Cockerell
7(6)	Metasomal pubescence entirely white	*Megachile brevis* Say
–	T3-T6 with mixed dark and light pubescence on discal surface	*Megachile pseudobrevis* Mitchell
8(5)	Mesonotum with white pubescence, no black hairs	*Megachile lippiae* Cockerell
–	Mesonotum with black hairs among white hairs	*Megachile texana* Cresson

## Species treatments

### Megachile (Litomegachile) brevis

Say, 1837

http://species-id.net/wiki/Megachile_brevis

Megachile brevis Say, 1837: 407. Syntypes male and female, USA: Indiana (destroyed).
Megachile lanuginosa Smith, 1853: 190. Syntypes male, female, USA: Florida (BMNH).
Megachile nupta Cresson, 1872: 268.Lectotype female, USA: Texas (USNM). *Megachile perbrevis* Cresson, 1878: 127. Lectotype male, USA: Texas (USNM).

#### Diagnosis.

**Figure 5.**
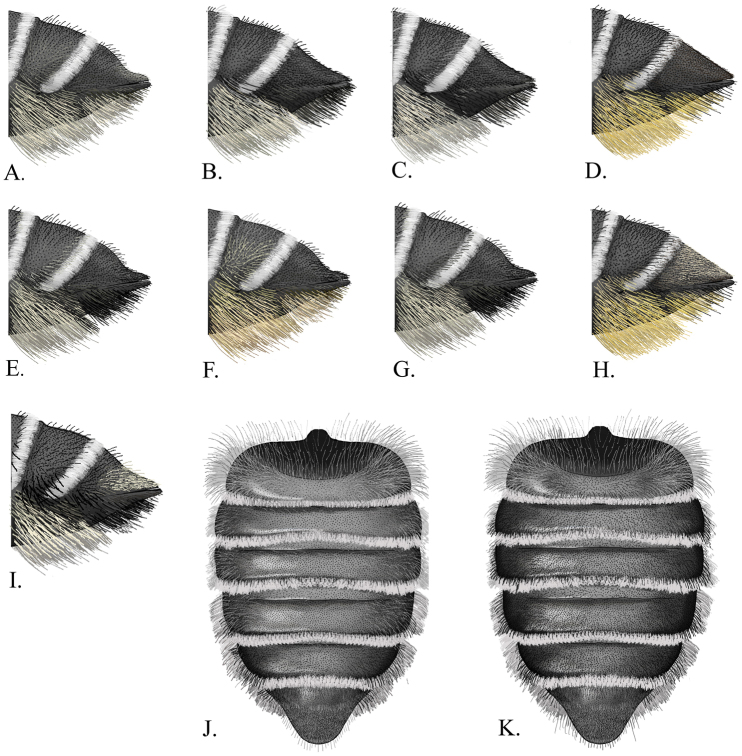
**A–I** Lateral view of T5-6 **A** *Megachile brevis* **B** *Megachile coquilletti* **C** *Megachile gentilis* **D** *Megachile mendica* **E** *Megachile onobrychidis* **F** *Megachile pankus* **G** *Megachile pseudobrevis* **H** *Megachile snowi* **I** *Megachile texana* **J–K** Dorsal view of metasoma **J** *Megachile lippiae* **K** *Megachile texana*.

*Megachile brevis* most closely resembles *Megachile onobrychidis*, *Megachile pseudobrevis*, and *Megachile coquilletti*. The female can be separated from these species by the combination of the ivory colored scopa, with a few black setae apically on S6, and with a small amount of white appressed pubescence apically on T6 (Figure 5A). The comparable species have more black setae and no white appressed pubescence on T6. The male has brown tarsi that distinguish it from *Megachile coquilletti* (Figure 4E), and a tomentum on T6 which distinguishes it from *Megachile onobrychidis*.

**Female.** Body length9–12 mm. Mandible 4-toothed, with no angulation between teeth 3 and 4 (Figure 4A). Head with white pubescence, vertex with black pubescence. Mesosoma with white pubescence, scutum with black pubescence. T2-3 with deep transverse basal grooves, T4 with shallow groove. T1 with white pubescence, T2 with white pubescence basally and black pubescence apically, T3-5 with black pubescence. T6 convex basally and concave apically in profile, and concave laterally in dorsal view; with black erect setae basally and black appressed pubescence, with some white appressed pubescence apically. S1-5 with ivory setae; S6 with ivory setae and few black setae apically (Figure 5A).

**Figure 6.**
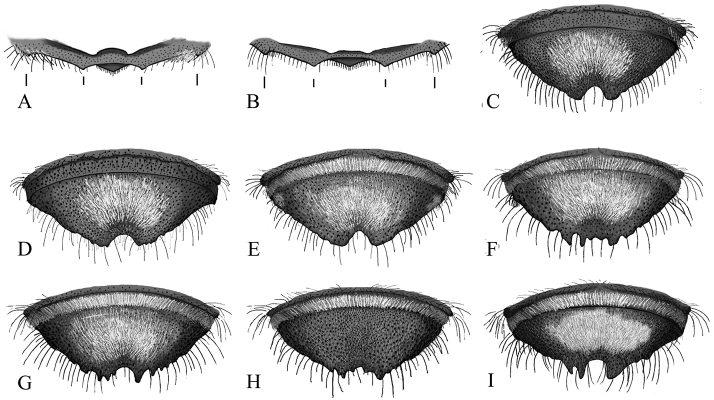
Male metasomal characters **A–B** Ventral view of true apical margin of T6 **A** Submedial teeth closer to each other than to lateral teeth, or distances equal **B** Submedial teeth closer to lateral teeth than each other **C–I** Male T6 posterior view **C** *Megachile gentilis* **D** *Megachile mendica* **E** *Megachile snowi* **F** *Megachile coquilletti* **G** *Megachile brevis* **H** *Megachile onobrychidis* **I** *Megachile lippiae*. **J, K** Metasoma dorsal view **J** *Megachile gentilis* **K** male *Megachile mendica*.

**Figure 7.**
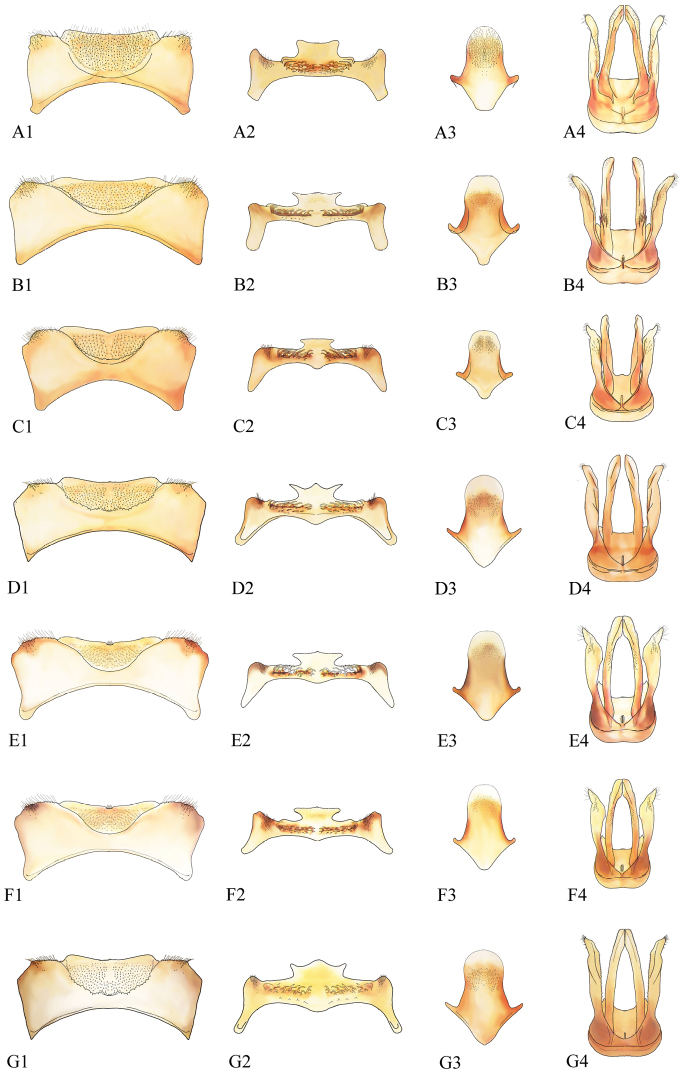
Male hidden sterna and genitalia. **A** *Megachile brevis*:1.S5 2.S6 3.S8 4. genitalia **B** *Megachile coquilletti*: 1. S5 2. S6 3. S8 4. genitalia **C** *Megachile gentilis*: 1. S5 2. S6 3. S8 4. genitalia **D** *Megachile lippiae*: 1. S5 2. S6 3. S8 4. genitalia **E** *Megachile mendica*: 1. S5 2. S6 3. S8 4. genitalia **F** *Megachile snowi*: 1. S5 2. S6 3. S8 4. genitalia **G** *Megachile texana*: 1. S5 2. S6 3. S8 4. genitalia.

**Male.** Body length7–9 mm. Mandible 3-toothed.Ocellocular distance equal to ocelloccipital distance (Figure 4D). Head with white pubescence. All mesosomal pubescence white or ivory (may appear yellow in early season specimens). T1-5 with white discal pubescence. T5 with complete apical fringe of white hair that covers marginal zone. T6 with tomentum (Figure 6G); transverse carina variable in shape, but usually with indistinct medial notch and asymmetrical jagged projections; true apical margin with submedial teeth closer to lateral teeth than each other (Figure 6B). Genitalia and hidden sterna shown in Figures 7A1–A4.

#### Variability.

The transverse carina of the male can vary significantly in this species, with some specimens barely showing any medial emargination, but most with jagged projections, where others have a medial notch.Females can have a few black scopal setae on S6 or all ivory colored scopae.

#### Distribution of material examined.

USA: California: Calaveras, Lake, Orange, Placer, Riverside, Sacramento, San Diego, San Joaquin, Siskiyou Tulare and Yolo Counties (May-Oct.); Colorado: Weld County (Sep.); Idaho: Bingham County (Jun.); Nebraska: Dawes County (Aug.); New Mexico: Eddy County (Oct.); Nevada: Churchill County (Jun); New York: Suffolk County (Aug.); Oklahoma: Marshall and Oklahoma Counties (Apr.); Oregon: Jackson County (Sep.); Texas: Gregg and Tyler Counties (Jun.-Sep.); Utah: Garfield and Washington Counties (Apr.-Sep.); 67 females, 68 males.

#### Ecology.

Michener (1953) published a detailed biology of *Megachile brevis* including a description of nest making, provisioning and development. *Megachile brevis* flies during the warmest parts of the year, with two to four generations per year, depending on locality and resources. It disperses widely from its natal site. Michener found that flower sources used by this species are diverse, but female bees tend to have a preference for blue, purple and white flowers, and a general faithfulness to a single type of pollen per collecting trip. M*egachile brevis* nested in a variety of situations, always nesting in preexisting hollows, including stems, burrows of other insects, dense foliage or spaces between rocks (Michener 1953). He also observed that *Megachile brevis* hunted for nesting sites by flying a few inches above the ground, and tended to nest near the soil surface. Larvae go through at least 4 instars (Baker et al. 1985). *Megachile brevis* nests are parasitized by a variety of species, including the megachilids *Coelioxys sayi* Robertson and *Coelioxys octodentata* Say, a clerid beetle (*Phyllobeanus* sp.), and wasps, including *Aprostocetus coelioxydis* Burks (Eulophidae), *Leucospis affinis affinis* Say (Leucospidae) and *Melittobia chalybii* Ashmead (Eulophidae) (Baker, 1985).

#### Flower records.

*Ailanthus* sp. (Simaroubaceae), *Amorpha canescens* (Fabaceae), *Baptisia* sp. (Fabaceae), *Cassia chamaecrista* (Fabaceae), *Centaurea jacea* (Asteraceae), *Erigeron philadelphicus* (Asteraceae), *Fagopyrum esculentum* (Polygonaceae), *Fallugia paradoxa* (Rosaceae), *Gossypium* sp. (Malvaceae), *Grindelia squarrosa* (Asteraceae), *Helianthus maximiliani* (Asteraceae), *Helianthus tuberosus* (Asteraceae), *Heliopsis scabra* (Asteraceae), *Kuhnistera purpurea* (Fabaceae), *Kuhnistera oligophylla* (Fabaceae), *Lactuca pulchella* (Asteraceae), *Machaeranthera tanacetifolia* (Asteraceae), *Marrubium vulgare* (Lamiaceae), *Medicago sativa* (Fabaceae), *Melilotus alba* (Fabaceae), *Melilotus officinalis* (Fabaceae), *Mentzelia* sp. (Loasaceae), *Meriolix serrulata* (Onagraceae), *Oxalis violacea* (Oxalidaceae), *Phyla incisa* (Verbenaceae), *Polygonum aubertii* (Polygonaceae), *Polygonum hydropiperoides* (Polygonaceae), *Psoralea floribunda* (Fabaceae), *Schrankia uncinata* (Fabaceae), *Solidago canadensis* (Asteraceae), *Solidago nemoralis* (Asteraceae), *Solidago rugosa* (Asteraceae), *Symphoricarpos occidentalis* (Caprifoliaceae), *Trifolium hybridum* (Fabaceae), *Vernonia baldwinii* (Asteraceae).

#### Comments.

**Figure 8.**
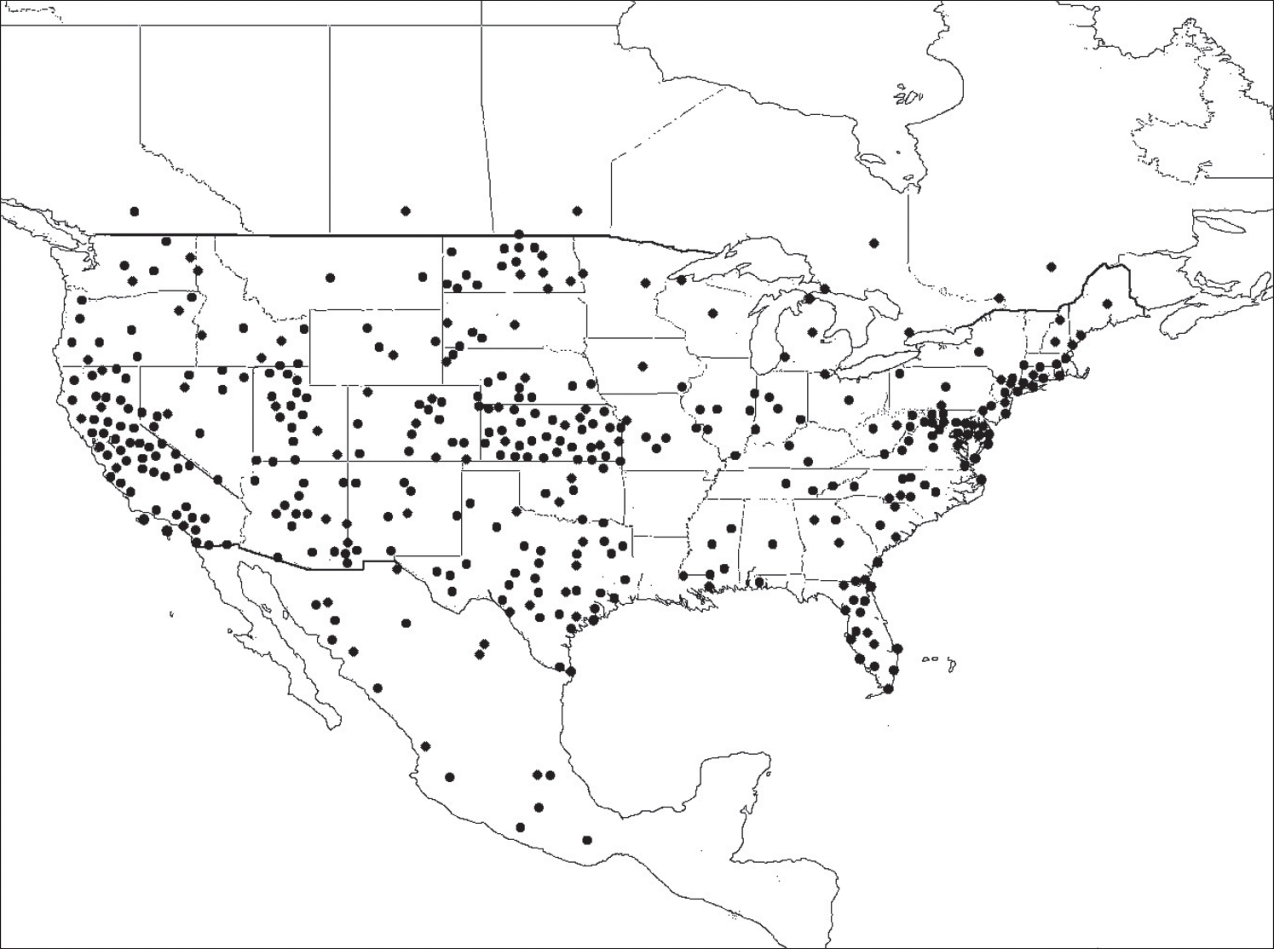
Distribution of *Megachile brevis*.

*Megachile brevis* is the type species of the subgenus *Litomegachile*. It ranges across North America, north to southern Saskatchewan, Canada, and south into Mexico. There is also a record from as far south as northern Costa Rica (not shown on map) (Ascher and Pickering 2011) (Figure 8).

### Megachile (Litomegachile) coquilletti

Cockerell, 1915

http://species-id.net/wiki/Megachile_coquilletti

Megachile mendica coquilletti Cockerell 1915: 535. Holotype male, USA: Texas (USNM).

#### Diagnosis.

Female *Megachile coquilletti* can be distinguished by the combination of a mandible with an even concavity in between teeth 3 and 4, and a slightly concave T6. It resembles *Megachile gentilis*, which has an angulation between teeth 3 and 4 of the mandible, and *Megachile brevis*, which has a much more concave T6 and much less black scopal setae on S6. Male *Megachile coquilletti* are easily distinguished from other *Litomegachile* by the foreleg with bicolored tarsomeres; the first 4 apical tarsomeres are yellow, contrasting with the darker basitarsus (Figure 4F). The males of all other species in the subgenus have uniformly brown foretarsi (Figure 4E).

**Female.** Body length11–12 mm. Mandible 4-toothed, with no angulation between teeth 3 and 4 (Figure 4A). T2-3 with deep transverse basal grooves, T4 with shallow groove. T1-5 with apical fringes of white hair that covers marginal zone; T1-2 with thin fringes of white hair, with white discal pubescence, T3-5 with black discal pubescence. T6 slightly concave in profile and laterally in dorsal view; with black appressed pubescence and black erect setae basally. S1-5 with ivory setae; S6 with some ivory setae basally, mostly black setae (Figure 5B).

**Male.** Body length9–12 mm. Mandible 3-toothed.Ocellocular distance less than ocelloccipital distance (Figure 4C). Foretarsus pale yellow, contrasting with darker basitarsus (Figure 4F). Head and mesosoma with white pubescence. T5 with apical fringe of white hair that covers marginal zone, interrupted medially. T6 with tomentum (Figure 6F); with transverse carina variable in shape, but usually with distinct medial notch and projections; true apical margin with submedial teeth closer to lateral teeth than each other (Figure 6B). Genitalia and hidden sterna shown in Figures 7B1–B4.

#### Variability.

Male tergal discal pubescence is variable in color. Some female specimens in fresh condition show a slight angulation between mandibular teeth 3 and 4. These may still be differentiated from *Megachile gentilis* by the lack of black setae on S5.

#### Distribution of material examined.

USA: California: El Dorado and Yolo Counties (Jun.-Aug.); Nevada: Clark, Humboldt and Lincoln Counties (May-Jul.); Texas: Fayetteville County (Sep.); Utah: Cache, Garfield and Washington Counties (May-Aug.); 42 females, 105 males.

#### Ecology.

*Megachile coquilletti* was collected in trap nests along the Cosumnes River south of Sacramento, California (Thorp et al. 1992).

#### Flower records.

*Asclepias speciosa* (Asclepiadaceae), *Cirsium vulgare* (Asteraceae), *Medicago sativa* (Fabaceae), *Polygonum aubertii* (Polygonaceae), *Salix* sp. (Salicaceae), *Salvia* sp. (Lamiaceae), *Solidago* sp. (Asteraceae), *Tamarix* sp. (Tamaricaceae).

#### Comments.

**Figure 9.**
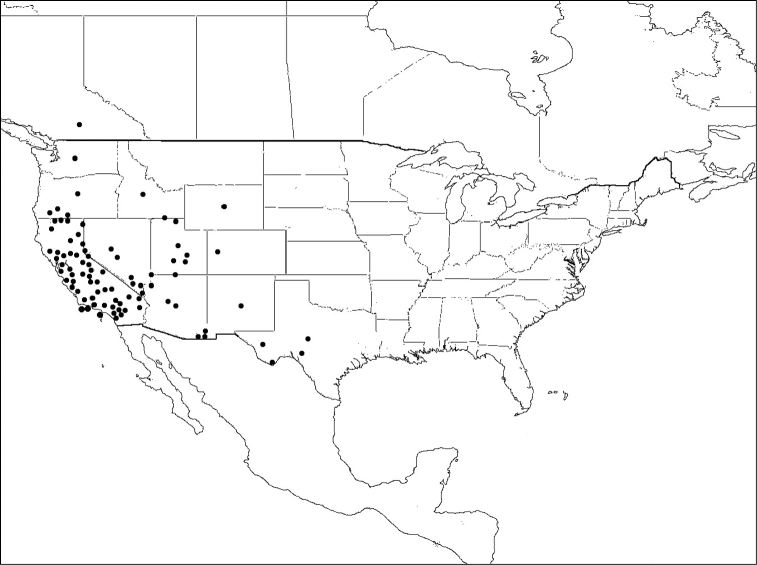
Distribution of *Megachile coquilletti*.

M.* coquilletti* is a western North American species (Figure 9).

### Megachile (Litomegachile) gentilis

Cresson, 1872

http://species-id.net/wiki/Megachile_gentilis

Megachile gentilis Cresson, 1872: 267. Holotype male, USA: Texas (ANSP).
Megachile palmarum Perkins, 1899: 114. Syntypes male female, USA: Hawaii (Repository?).
Megachile murinella Cockerell, 1908: 263. Holotype female, USA: New Mexico (USNM).

#### Diagnosis.

*Megachile gentilis* closely resembles *Megachile mendica*. The males of the two species can only be separated by two characters. In *Megachile gentilis*, the punctures on T6 are nearly contiguous creating the appearance of small ridges, with shiny surface almost completely obscured, and T2 has an apical fringe of white hair, while the fringe is absent in *Megachile mendica*. The females are slightly easier to differentiate. *Megachile gentilis* has a very slightly concave S6, with black pubescence and some erect setae basally. *Megachile mendica* has brown appressed pubescence and no erect setae. Also, *Megachile gentilis* has black scopal setae on S6 and basally on S5, while *Megachile mendica* has black setae only apically on S6. *Megachile gentilis* females also resemble *Megachile coquilletti* females. These can be differentiated by the angulate mandible of *Megachile gentilis* (Figure 4B).

**Female.** Body length 11–12 mm. Mandible 4-toothed, with surface between teeth 3 and 4 angulate (Figure 4B). T2-3 with deep transverse basal grooves, T4 with shallow groove. T1-5 with apical fringes of white hair that covers marginal zone, T1-2 with thin fringes of white hair. T1-2 with white discal pubescence, T3-5 with black discal pubescence. T6 very slightly concave in profile and laterally in dorsal view; with black appressed pubescence and black erect setae basally. (Figure 5C). S1-5 with ivory setae; S6 with black setae.

**Male.** Body length 9–11 mm.Mandible 3-toothed.Ocellocular distance less than ocelloccipital distance (Figure 4C). Head with white pubescence; vertex with black pubescence. Mesosoma with white pubescence, scutum with black pubescence. T2 with thin apical fringe of white hair (Figure 6J). T5 without complete apical fringe of white hair that covers marginal zone, may have some hair laterally. T6 with tomentum; punctures crowded, nearly contiguous (Figure 6C); transverse carina with distinct medial notch; true apical margin with submedial teeth closer to each other than lateral teeth, or distances equal (Figure 6A). Genitalia and hidden sterna shown in Figures 7C1–C4.

#### Variability.

As with other *Litomegachile* species, individuals that appear early in the flight season may have pubescence that appears yellow instead of white.

#### Distribution of material examined:

USA: Arizona: Cochise, Pima and Santa Cruz Counties (Apr-Sep); California: Contra Costa, Mariposa Mendocino, Tuolumne and Yolo Counties (Jun.-Sep.); Utah: Washington County (May); Texas: Brewster County (May). MEXICO: Chihuahua and Sonora (Sep.); 103 females, 188 males.

#### Ecology.

*Megachile gentilis* will nest in trap nests. Krombein (1967) recovered nests from trap nests placed under live or dead mesquite branches in open desert. Parasites reared by Krombein (1967) from these traps included *Tetrastichus megachilidis* Burks (Eulophidae), *Trichodes horni* Wolcott & Chapin (Cleridae), *Anthrax atriplex* Marston (Bombyliidae), and *Anthrax irroratus* Say(Bombyliidae).

#### Flower records.

*Clarkia biloba* (Onagraceae), *Eriodictyon* sp. (Boraginaceae), *Gaillardia pulchella* (Asteraceae), *Melilotus alba* (Fabaceae), *Parkinsonia* sp. (Fabaceae), *Polygonum aubertii* (Polygonaceae).

#### Comments.

**Figure 10.**
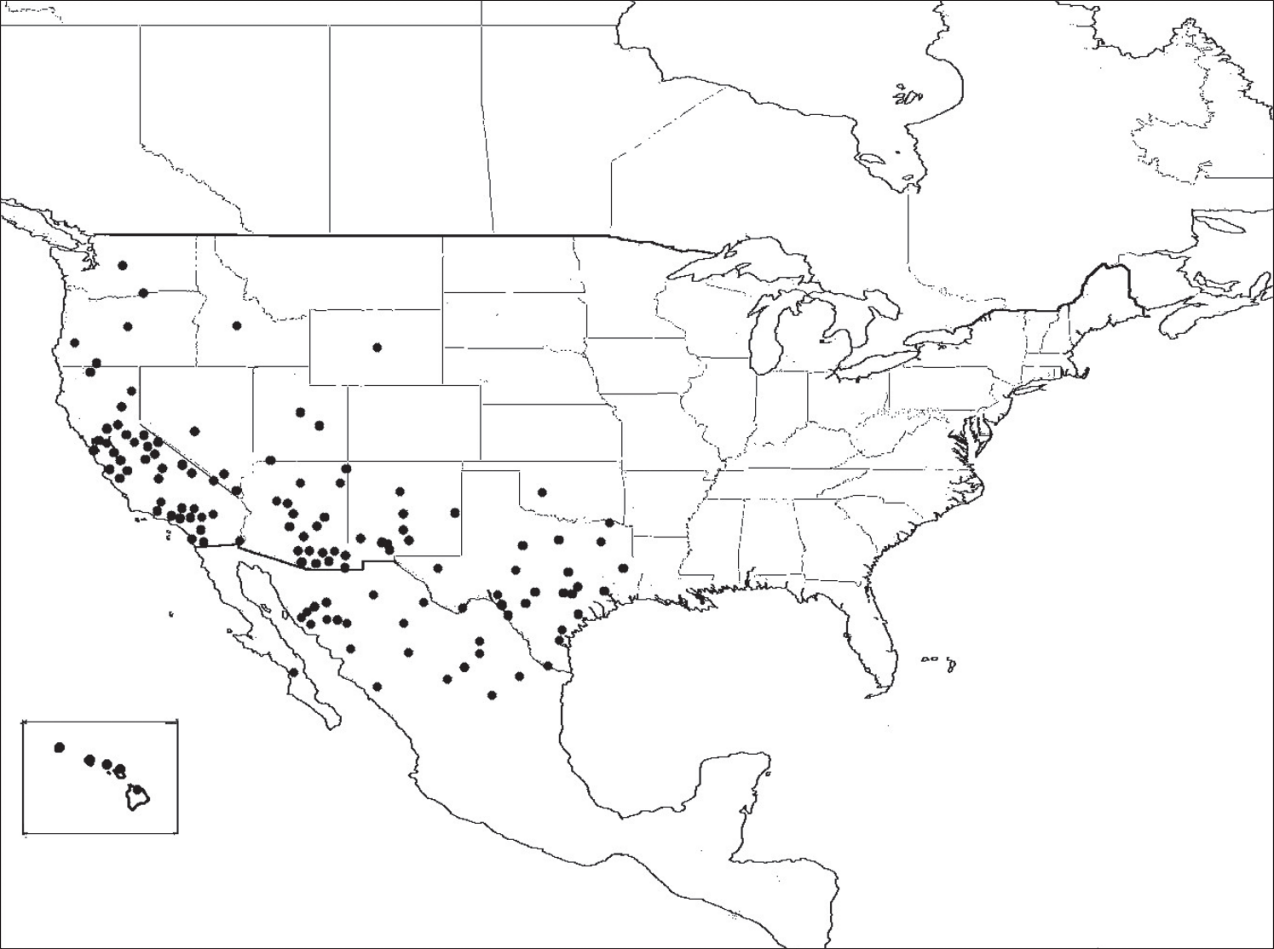
Distribution of *Megachile gentilis*.

*Megachile gentilis* is a western North American species, though records occur from eastern Texas, and populations are established in Hawaii (Snelling 2003) (Figure 10).

### Megachile (Litomegachile) lippiae

Cockerell, 1900

http://species-id.net/wiki/Megachile_lippiae

Megachile cleomis var. lippiae Cockerell, 1900: 15. Holotype female, USA: New Mexico (CAS).
Megachile schismatura Cockerell, 1908: 267. Lectotype female, USA: New Mexico (USNM). New synonymy.

#### Diagnosis.

Female *Megachile lippiae* are distinguished from *Megachile texana* by looking at features of the metasoma in dorsal view. *Megachile lippiae* has black setae laterally only on T5-6 and sometimes a few black setae on T4 (Figure 5J). *Megachile texana* has some black setae on all tergal segments. The male *Megachile lippiae* has no black pubescence except sometimes on the vertex of the head. *Megachile texana* has black pubescence on the vertex of the head and the center of the mesonotum.

**Female.** Body length12–14 mm. Mandible 4-toothed, with no angulation between teeth 3 and 4 (Figure 4A). T2-4 with deep transverse basal grooves. T1-5 with apical fringes of white hair that covers marginal zone; T1 with thin apical fringe of white hair . T1-4 with white discal pubescence, T5-6 with black setae apparent laterally in dorsal view (Figure 5J). T6 deeply and evenly concave in profile and laterally in dorsal view; with black erect setae basally and white appressed pubescence apically. S1-4 with ivory setae; S5 with ivory setae basally, black setae apically; S6 with black setae.

**Male.** Body length11–13 mm. Mandible 3-toothed.Ocellocular distance less than ocelloccipital distance (Figure 4C). All pubescence white (may appear yellow in early season specimens). T5 with complete apical fringe of white hair that covers marginal zone. T6 with tomentum; transverse carina with deep distinct medial notch and fingerlike projections (Figure 6I); true apical margin with submedial teeth closer to lateral teeth than each other (Figure 6B). Genitalia and hidden sterna shown in Figure 7D1–D4.

#### Variability.

Male tergal discal pubescence is variable in color. Body hair may appear yellow in early season individuals. Females can have black setae that occur laterally on T4.

#### Distribution of material examined.

USA: Arizona: Cochise, Santa Cruz and Yavapai Counties (Aug.-Sep.); California: Los Angeles, Riverside and Yolo Counties (Jun.-Sep.); New Mexico: Hidalgo County (Aug.); 59 females, 68 males.

#### Flower records.

*Asclepias* sp. (Asclepiadaceae),*Cevallia sinuata* (Loasaceae), *Eriodictyon angustifolium* (Boraginaceae),*Larrea tridentata* (Zygophyllaceae),*Lupinus* sp. (Fabaceae),*Melilotus alba* (Fabaceae),*Prosopis* sp.(Fabaceae), *Verbesina encelioides* (Asteraceae).

#### Comments.

**Figure 11.**
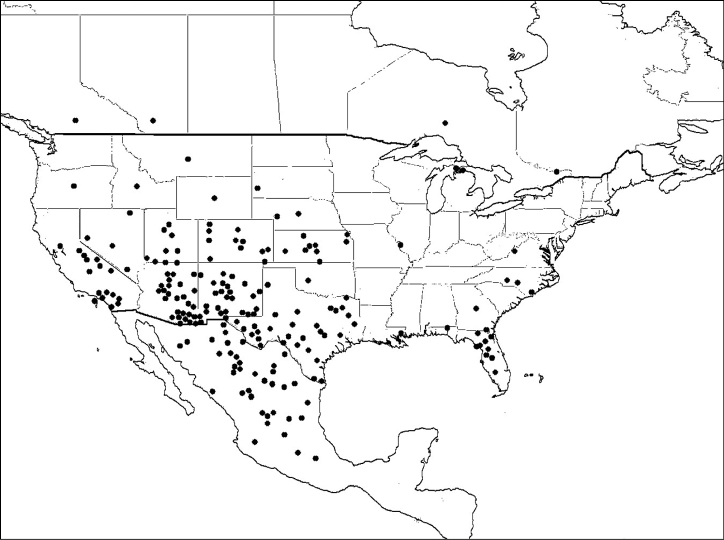
Distribution of *Megachile lippiae*.

*Megachile lippiae* was originally described as a subspecies of *Megachile texana* (Mitchell, 1935). It was raised to species level by Sheffield et al. (2011). *Megachile lippiae* is primarily a western North American species, though records exist from eastern localities (Figure 11). *Megachile schismatura* is removed from synonymy under *Megachile texana* and placed as a synonym of *Megachile lippiae* herein.

### Megachile (Litomegachile) mendica

Cresson, 1878

http://species-id.net/wiki/Megachile_mendica

Megachile mendica Cresson, 1878: 126 Holotype female, USA: California (ANSP).

#### Diagnosis.

*Megachile mendica* closely resembles *Megachile gentilis*. The females can be distinguished by difference in the T6 structure and pubescence color, and scopa color. Female *Megachile mendica* have a very straight T6 in profile, and slightly concave laterally in dorsal view. The appressed pubescence on T6 is brownish in color. The scopa is yellowish, distinguishing it from other *Litomegachile* females which have a pale ivory colored scopa. An exception is *Megachile pankus*, which also has a yellow scopa, but it can be separated by its concave T6 in contrast with the straight T6 of *Megachile mendica*. The male *Megachile mendica* can be distinguished from *Megachile gentilis* by the distance between punctures on T6. *Megachile mendica* punctures occur roughly 0.25–0.5 the width of a puncture apart so that you can see the shiny discal surface in between (Figure 6D) Male *Megachile mendica* also lack the apical fringe of white hair on T2. Males of other species of *Litomegachile* have a complete apical fringe of white hair on T2.

**Female.** Body length11–13 mm. Mandible 4-toothed, with surface between teeth 3 and 4 angulate (Figure 4B). T2-4 with shallow transverse basal grooves. T1-5 with apical fringes of white hair that covers marginal zone; T1-2 with medially interrupted fringes of white hair. T1 with white discal pubescence; T2-5 with black discal pubescence. T6 straight in profile and slightly concave laterally in dorsal view; with brown appressed pubescence, without erect setae. S1-5 and 6 with yellow setae, S6 with black setae apically (Figure 5D).

**Male.** Body length 8–10 mm. Mandible 3-toothed**.** Ocellocular distance less than ocelloccipital distance (Figure 4C) Head with white pubescence, vertex with black pubescence. Mesosoma with white pubescence, scutum with black pubescence. T1-2 pubescence white; T3-5 white pubescence basally, black pubescence apically. T2 without thin apical fringe of white hair (Figure 6K). T5 without complete white hair fringe that covers marginal zone; may have some hair laterally. T6 punctures separated; shiny discal surface visible between; with tomentum; transverse carina with a distinct medial notch (Figure 6D); true apical margin with median teeth closer to each other than to lateral teeth, or distances equal (Figure 6A). Genitalia and hidden sterna shown in Figures 7E1–E4.

#### Distribution of material examined.

USA: Arkansas: Pulaski County (Sep.); Delaware: New Castle County; Florida:Alachua and Monroe Counties (Jul.-Aug.); Georgia: Liberty County (Jun.); Illinois: Cook County (Aug.); Kansas: Douglas County (Aug.); Kentucky: Wayne County (Jul.); Maryland: Anne Arundel and Montgomery Counties (Jul.-Sep.); Missouri: Lapeer County(Jul); Mississippi: Oktibbeha County (Jun.); North Carolina: Pender County (Sep.); New Jersey: Atlantic and Burlington Counties (May.-Aug.); New York: Kings and Westchester Counties(Aug.); Oklahoma: Marshall County (Apr.); South Carolina: Chesterfield County (Sep.); Texas: Maverick County (May); Virginia: Clarke, Loudoun, Page and Shenandoah *Counties* (Jul.); West Virginia: Hampshire County (Jul.); Washington D.C. (Jun.-Oct.); 25 females, 42 males.

#### Ecology.

*Megachile mendica* seems to be flexible in its choice of nesting sites across different habitats. When it nests in trap nests, it prefers a cavity diameter of around 8 mm, which is also preferred by *Megachile brevis* (Baker et al. 1985). In Texas, *Megachile mendica* was found to nest in sandy soil, and like *Megachile texana*, it will also excavate burrows in the soil (Williams et al. 1986). Krombein (1967) reared *Megachile mendica* from wooden block traps placed on limbs of pine oak and hickory. Generation number and times differed based on the locality (Krombein 1967). Medler (1965) reared *Megachile mendica* at 21 degrees Celsius and found that they went from egg to mature larva in one week, spun a cocoon in one day, and took about 3 weeks for pupal development and adult emergence. An *Megachile mendica* larva was illustrated and described by Baker et al. (1985). In addition to *Coelioxys* sp. and *Leucospis affinis affinis* (Leucospidae), *Megachile mendica* nests are known to be parasitized by the flies *Anthrax irroratus irroratus* (Bombyliidae) and *Megaselia* sp.(Phoridae) (Baker et al. 1985).

#### Flower records.

*Amorpha fruticosa* (Fabaceae), *Aster paniculatus* (Asteraceae), *Balduina angustifolia* (Asteraceae), *Bidens alba* (Asteraceae), *Calamintha ashei* (Lamiaceae), *Centaurea jacea* (Asteraceae), *Cephalanthus occidentalis* (Rubiaceae), *Chrysanthemum leucanthemum* (Asteraceae) *Pityopsis graminifolia* (Asteraceae), *Conoclinium coelestinum* (Asteraceae), *Eupatoriadelphus maculatus* (Asteraceae), *Flaveria linearis* (Asteraceae), *Helenium amarum* (Asteraceae), *Helianthus divaricatus* (Asteraceae), *Helianthus tuberosus* (Asteraceae), *Lavandula dentata* (Lamiaceae), *Medicago sativa* (Fabaceae), *Melilotus alba* (Fabaceae), *Parthenocissus quinquefolia* (Vitaceae), *Phaseolus* sp. (Fabaceae), *Psoralea floribunda* (Fabaceae), *Polygonum hydropiperoides* (Polygonaceae), *Rhus glabra* (Anacardiaceae), *Rubus* sp. (Rosaceae), *Silybum* sp. (Asteraceae), *Solidago serotina* (Asteraceae), *Tephrosia virginiana* (Fabaceae), *Vicia floridana* (Fabaceae).

#### Comments.

**Figure 12.**
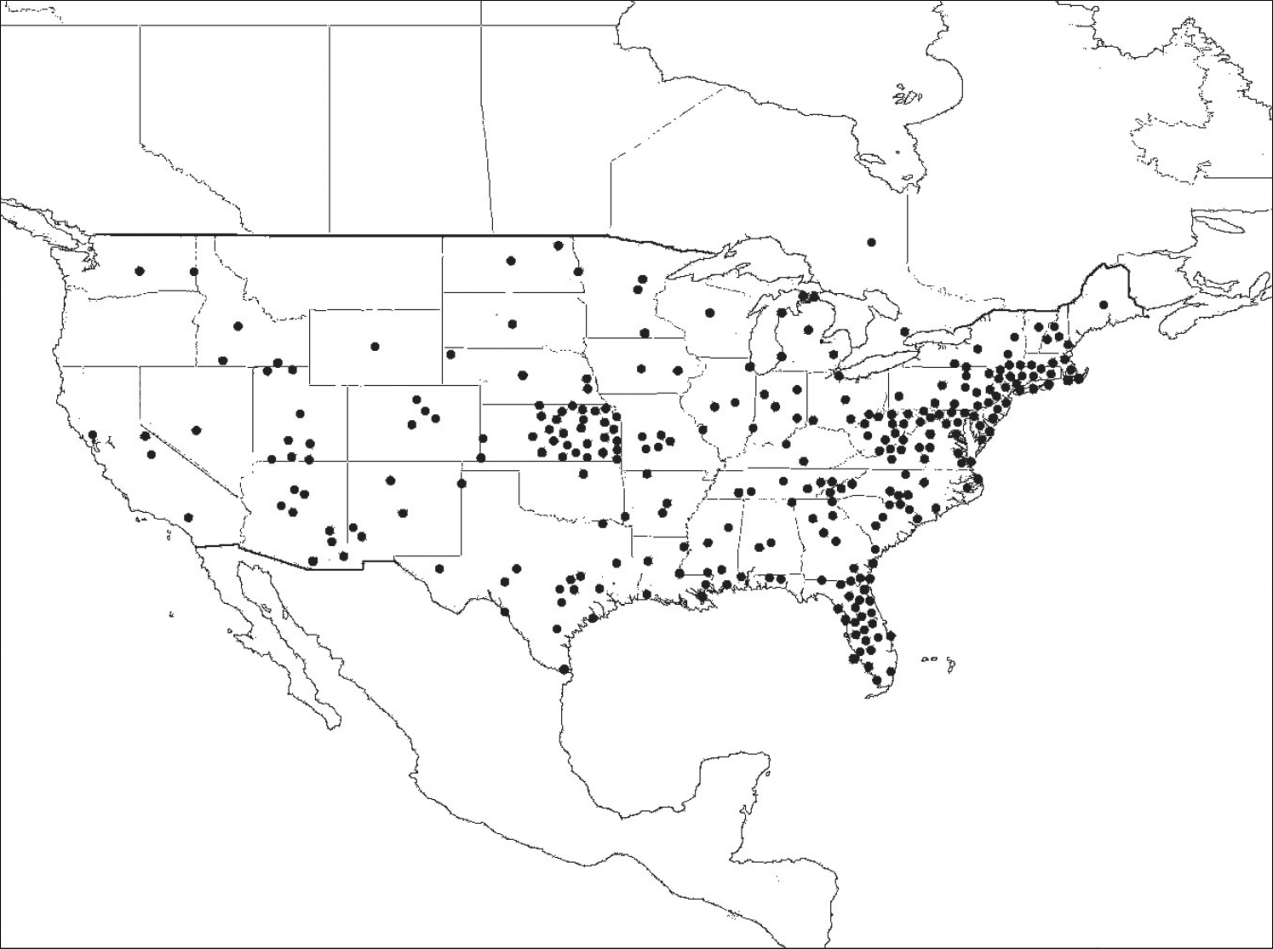
Distribution of *Megachile mendica*.

*Megachile mendica* is distributed across North America south to Zacatecas, Mexico, though it was considered more of an eastern species by Mitchell (1934) (Figure 12).

### Megachile (Litomegachile) onobrychidis

Cockerell, 1908 

http://species-id.net/wiki/Megachile_onobrychidis

Megachile onobrychidis Cockerell, 1908: 266 Holotype male, USA: New Mexico (CAS).

#### Diagnosis.

The male *Megachile onobrychidis* is best distinguished from other species in this subgenus by the lack of a white tomentum on T6.The female *Megachile onobrychidis* resembles *Megachile brevis*, but with entirely black setae on S6 and apically on S5, and no pale appressed pubescence on T6.

**Female.** Body length9–12 mm. Mandible 4-toothed with no angulation on surface between teeth 3 and 4 (Figure 4A). T2-3 with deep transverse basal groove, T4 with shallow groove. T1-5 with apical fringes of white hair that covers marginal zone; T1-2 with thin or medially interrupted fringes of white hair, and white discal pubescence; T3-5 with black discal pubescence. T6 convex basally and concave apically in profile, and concave laterally in dorsal view; with erect setae basally and black appressed pubescence. S1-4 with ivory setae; S5 with black setae apically, ivory setae basally; S6 with black setae (Figure 5E).

**Male.** Body length7-9 mm. Mandible 3-toothed. Ocellocular distance equal to ocelloccipital distance (Figure 4D). T1-2 with white discal pubescence; T4-6 with white discal pubescence basally, black pubescence apically. Head and mesosoma with white pubescence (may appear yellow in early season specimens). T5 with complete fringe of white hair that covers marginal zone. T6 without tomentum, hairs sparse and discal surface clearly visible beneath (Figure 6H); transverse carina variable in shape, usually with indistinct medial notch and asymmetrical jagged projections; true apical margin with submedial teeth closer to lateral teeth than each other (Figure 6B). Genitalia and hidden sterna resemble those of *Megachile brevis* (Figures 7A1-A4).

#### Variability.

Male *Megachile onobrychidis* are separated from *Megachile brevis* in part by the lack of a tomentum on T6. Some specimens have no tomentum while others have sparse tomentum type hairs, but as long as these hairs are sparse enough so that the tergal surface is still visible, they are *Megachile onobrychidis*.

#### Distribution of material examined.

USA: Arizona: Cochise County (Aug.); California: Calaveras, Colusa Contra Costa, Humboldt, Imperial, Lake, Lassen, Los Angeles, Mendocino, Merced, Modoc, Monterey, Napa, Nevada, Orange, Placer, Plumas, Riverside, Sacramento, San Bernardino, Shasta, Siskiyou, Sonoma, Stanislaus, Tehama, Tuolumne, Tulare, Yolo and Yuba Counties (May-Oct.); Idaho: Canyon County (Aug.); Nevada: Churchill, Elko, Humboldt, Lyon and Washoe Counties (Jun.-Aug.); Oregon: Cassia and Jackson Counties (Jun.-Jul.); Utah: Cache and Wasatch Counties (Jun.-Aug.); MEXICO: Sinaloa, Sonora. 126 females, 193 males.

#### Flower records.

*Asclepias speciosa* (Asclepiadaceae), *Calothamnus* sp. (Myrtaceae), *Clarkia biloba* (Onagraceae), *Clarkia dudleyana* (Onagraceae), *Clarkia unguiculata* (Onagraceae), *Dalea polydenia* (Fabaceae), *Daucus* sp.(Apiaceae), *Grindelia camporum* (Asteraceae), *Lactuca pulchella* (Asteraceae), *Mentzelia* sp. (Loasaceae), *Phacelia* sp. (Hydrophyllaceae), *Polygonum aubertii* (Polygonaceae).

#### Comments.

**Figure 13.**
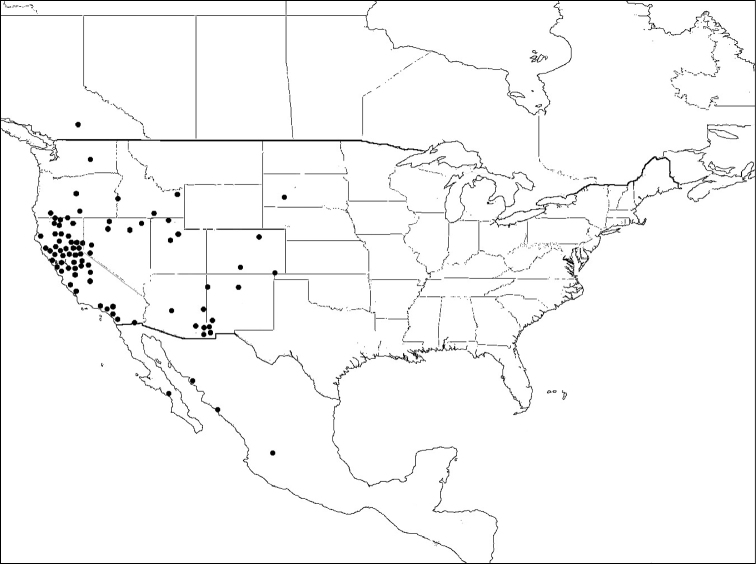
Distribution of *Megachile onobrychidis*.

Mitchell (1935) listed this species as a subspecies of *Megachile brevis*. It was elevated to species level by Sheffield et al. (2011). It is a western North American species extending south to Sinaloa, Mexico. (Figure 13).

### Megachile (Litomegachile) pankus sp. n.

urn:lsid:zoobank.org:act:80ED5270-BA6B-42C8-AB4B-AEFA7A531D7A

http://species-id.net/wiki/Megachile_pankus

#### Type material.

Holotype female: MEXICO: Hidalgo, Pachuca, 11 Jun 1935, R. M. and G. E. Bohart (BBSL). Paratypes: 1 female: MEXICO: Sonora, Alamos, 4 Sep 1991 (AMNH), 1 female: MEXICO: Sinaloa, Mazatlan 28 Oct 1969 (BBSL); 1 female: MEXICO: Sinaloa, 4 mi NW Choix, 31 Aug 1968 (BMEC); 1 female: MEXICO: Sinaloa, 6 mi NW Choix, 6 Aug 1968 (BMEC).

#### Diagnosis.

*Megachile pankus* is unique among *Litomegachile* species because the female has a mandible with an angulation between teeth 3 and 4, and T6 is basally convex and apically concave. No other species in the subgenus has this combination of characters. The female *Megachile onobrychidis* has similar metasomal features, but has more black setae on S6, while *Megachile pankus* has only a few black setae on T6. It can also be further distinguished from *Megachile onobrychidis* and *Megachile brevis* by the angulation between teeth 3 and 4 of the mandible. T6 is convex basally and concave apically in profile, and concave laterally in dorsal view, which distinguishes it from *Megachile mendica* or *Megachile gentilis*.

#### Female description.

Body length 10 mm. Forewing length 7 mm. Head: HL 0.7× HW; compound eyes convergent below, with upper inner margins slightly convergent above; lateral ocelli closer to margin of vertex than edge of compound eye; compound eye width 1.1× width of genal area in lateral view; clypeus twice as wide as high (Figure 3B); clypeus and supraclypeal area slightly convex; punctation fine, with larger punctures on clypeus, becoming smaller on supraclypeal, paraocular area, vertex and rest of head; punctures never separated by more than 0.3× puncture diameter; labrum width 0.8× length; AD 3× width of antennal socket, ID 0.6× ASO; ID 1.36× length of scape; mandible with recessed cutting edges between teeth 3 and 4 and incomplete recessed cutting edge that forms rough right angle between teeth 3 and 2; surface between teeth 3 and 4 angulate (Figure 2C, 4B); scape length 4.3× width, with white setae; pedicel and F1 width 0.8× length; pedicel length 0.8× F1; F2-6 length equal to width; F7-8 length 0.9× width; F9 length 0.8× width; F10 length 0.7× width; Mesosoma: mesepisternum convex, large and pronounced, twice as wide as pronotum; scutum length 0.8× width; scutellum length 0.3× scutum length, scutellum width 0.4× scutellum length; tegula twice as long as wide; scutum 7.2× width of tegula. Wings: forewing length 2.7× width; WCL 0.8 × length of wing; SL 0.2 × MCL; with two submarginal cells, first submarginal crossvein angled parallel to medial vein, second submarginal crossvein angulate; distance from distal edge of stigma to wing base 0.7× distance from wing base to distal edge of marginal cell; hindwing with jugal lobe that does not extend past cubital cell; LTJ 0.3× HWL; LTV 0.5 × HWL (Figure 3A). Legs: ratio of segment length of foreleg (compared to FL): CL 0.6×, TL 0.3×, FL 1×, TBL 0.9×, TRL 1.2×, BTL 0.5×, DTL 0.3×; foreleg with tibial spur modified as antennal cleaner, TSL 0.2×; midleg segment ratios: CL 0.7×, TL 0.4×, FL 1×, TBL 1×, TRL 1.3×, BTL 0.7×, DTL 0.3×; foreleg with tibial spur, TSL 0.3× TBL; hindleg segment ratios: CL 0.5×, TL 0.3×, FL 1×, TBL 0.9×, TRL 1.4×, BTL 0.7×, DTL 0.3×; tibia with two spurs, TSL 0.4× TBL; hindleg with basitarsus dilated 4.5× width of distitarsus (Figure 3C). Metasoma: T2-4 with shallow transverse basal grooves; T1-5 with apical fringes of white hair covering marginal zone, T1-2 fringe widths 0.2× width of discal surface medially, T3-5 fringe widths 0.3a width of discal surface medially; T1-2 apical fringes of white hair more sparse, marginal zone slightly visible between hairs; T1-5 with white discal pubescence; T6 discal surface with black appressed pubescence and black erect setae; T6 convex basally and concave apically in profile, and concave laterally in dorsal view;S1-5 with yellow setae; S6 with yellow setae and some black setae apically (Figure 5F). Color: Body black, legs brownish distally, wing membrane slightly tinted brown, veins brown (Figure 2A-B). Pubescence: White on head except ocellar region black; paraocular area, supraclypeal area and clypeus with dense pubescence obscuring view of integument; vertex with sparse pubescence with integument visible beneath; genal area with pubescence sparse beginning at dorsal surface, progressively more dense toward malar area. Mesosomal pubescence white; dense around tegula and behind scutellum, sparse on scutum, dense on ventral mesosomal surface.

#### Male.

unknown

#### Etymology.

The species name ‘*pankus’* is a nonsense combination.

#### Distribution.

**Figure 14.**
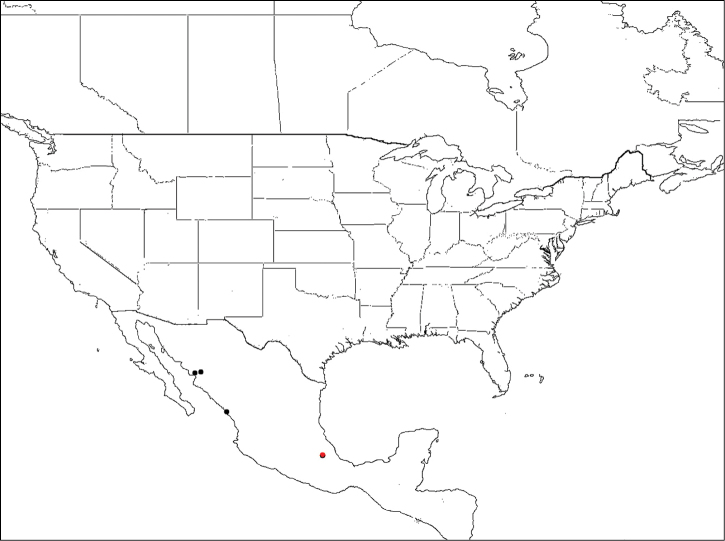
Distribution of *Megachile pankus* (red indicates locality of holotype).

*Megachile pankus* has only been collected in Mexico (Figure 14).

#### Flower records.

*Petalostemon* sp. (Fabaceae).

### Megachile pseudobrevis

Mitchell, 1934

http://species-id.net/wiki/Megachile_pseudobrevis

Megachile brevis pseudobrevis Mitchell, 1934 Holotype female, USA: Florida (NCSU).

#### Diagnosis.

*Megachile pseudobrevis* closely resembles *Megachile brevis* and *Megachile onobrychidis*. The differences between *Megachile pseudobrevis* and *Megachile brevis* are slight. Female *Megachile pseudobrevis* has less black appressed pubescence on T6 than *Megachile brevis*. Also the scopa of *Megachile pseudobrevis* has less black setae than *Megachile onobrychidis*, with black setae being restricted to S6. *Megachile pseudobrevis* has more black setae than *Megachile brevis*, which has often only a few black setae apically on S6.

**Female.** Body length9–11 mm. Mandible 4-toothed, with no angulation between teeth 3 and 4 (Figure 4A). T2-3 with deep transverse basal groove, T4 with shallow basal groove. T1-5 with apical fringes of white hair covering marginal zone; T1-2 with medially interrupted fringes of white hair. T1 with white discal pubescence; T2 discal pubescence white basally, black apically; T3-5 with black discal pubescence. T6 convex basally and concave apically in profile, concave laterally in dorsal view; with black erect setae basally and black appressed pubescence. S1-5 with ivory setae; S6 with black setae (Figure 5G).

**Male.** Body length7–9 mm. Mandible 3-toothed.Ocellocular distance equal to ocelloccipital distance (Figure 4D). T5 with complete apical fringe of white hair covering marginal zone. T6 with tomentum; transverse carina variable in shape, usually with indistinct medial notch and asymmetrical jagged projections; true apical margin with submedial teeth closer to lateral teeth than each other (Figure 6B). Genitalia and hidden sterna resemble those of *Megachile brevis* (Figures 7A1–A4).

#### Distribution of material examined.

USA: Florida: Alachua, Duval, Monroe and Orange Counties (Mar.-Sep.); 14 females, 16 males.

#### Ecology.

Packer (1987) observed *Megachile pseudobrevis* nesting in tufts of grass, creating nests of single cells. *Megachile pseudobrevis* preferred the commonest flowering plant *Bidens pilosa* (Asteraceae) at the site as a source for cutting nesting material, but also used petals from *Eustoma exaltatum* (Gentianaceae).Nests were parasitized by the meloid beetle *Nemognatha punctulata* LeConte (Packer 1987).

#### Flower records.

*Balduina angustifolia* (Asteraceae), *Bidens pilosa* (Asteraceae), *Eriogonum tomentosum* (Polygonaceae), *Eustoma exaltatum* (Gentianaceae), *Lupinus cumulicola* (*Fabaceae*), *Vitex agnus castus* (Verbenaceae).

#### Comments.

**Figure 15.**
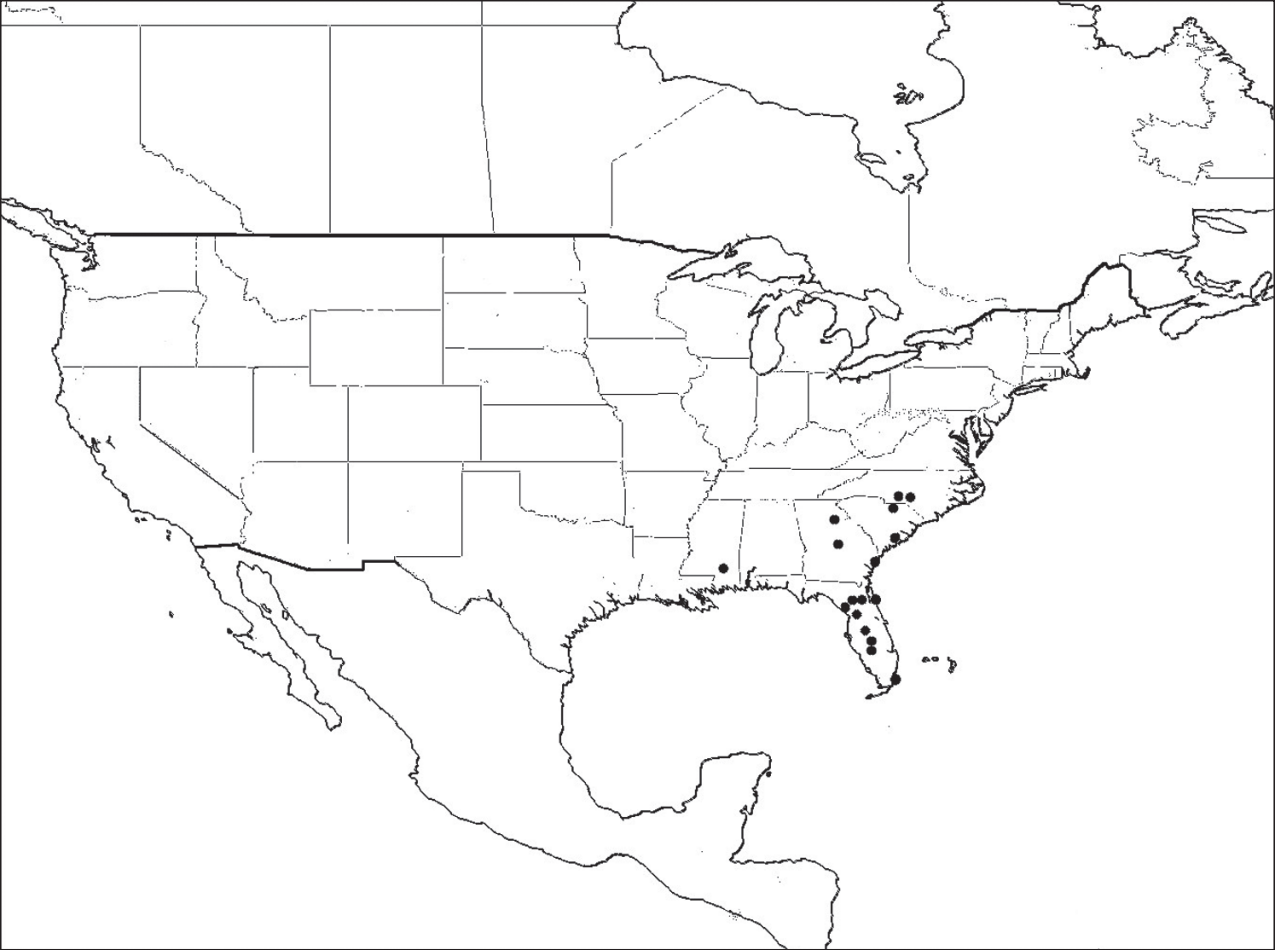
Distribution of *Megachile pseudobrevis*.

*Megachile pseudobrevis* was originally described as a variety of *Megachile brevis*. It was raised to species level by Sheffield et al. (2011). This species has a limited range occurring in the southeastern United States (Figure 15).

### Megachile (Litomegachile) snowi

Mitchell, 1927 stat. n.

http://species-id.net/wiki/Megachile_snowi

Megachile mendica snowi Mitchell, 1927:113 Holotype female, USA: Arizona (MCZ).

#### Diagnosis.

*Megachile snowi* is distinguished from *Megachile mendica* in males by the presence of a complete apical fringe of white hair on T5. *Megachile mendica* has little or no apical fringe of white hair on T5. Female *Megachile snowi* have white appressed pubescence on T6, and the few black scopal setae of S6 are only found apically. *Megachile mendica* has brown pubescence on T6, and S6 has more black setae.

**Female.** Body length 11–13 mm. Mandible 4-toothed, with surface between teeth 3 and 4 angulate (Figure 4B). T2-4 with shallow transverse basal groove. T1-5 with apical fringes of white hair covering marginal zone; T1-2 with medially interrupted fringes of white hair. T1-2 with white discal pubescence; T3-5 with black discal pubescence. T6 straight in profile and slightly concave laterally in dorsal view; without erect setae, with white appressed pubescence. S1-5 with yellow setae; S6 with yellow setae and few black setae apically (Figure 5H).

**Male.** Body length 8–10 mm. Mandible 3-toothed.Ocellocular distance less than ocelloccipital distance (Figure 4C). Mesosoma with white pubescence. T1-3 with white discal pubescence; T4-5 with white pubescence basally, black apically. T2 with thin apical fringe of white hair. T5 with complete apical fringe of white hair covering marginal zone. T6 with tomentum (Figure 6E);transverse carina with a distinct medial notch; true apical margin with submedial teeth closer to each other than to lateral teeth, or distances equal (Figure 6A). Genitalia and hidden sterna shown in Figures 7F1–F4.

#### Distribution of material examined.

USA: Arizona: Cochise County (Aug.-Sep.); California: *Mariposa County* (May); Colorado: Boulder County (May-Jun.); New Mexico: Catron County (Jul.); Utah: Cache, Garfield, Kane and Salt Lake Counties (May-Aug.); MEXICO: Zacatecas.17 females, 35 males.

#### Flower records.

*Cirsium* sp. (Asteraceae), *Helianthus* sp. (Asteraceae), *Melilotus alba* (Fabaceae).

#### Comments.

**Figure 16.**
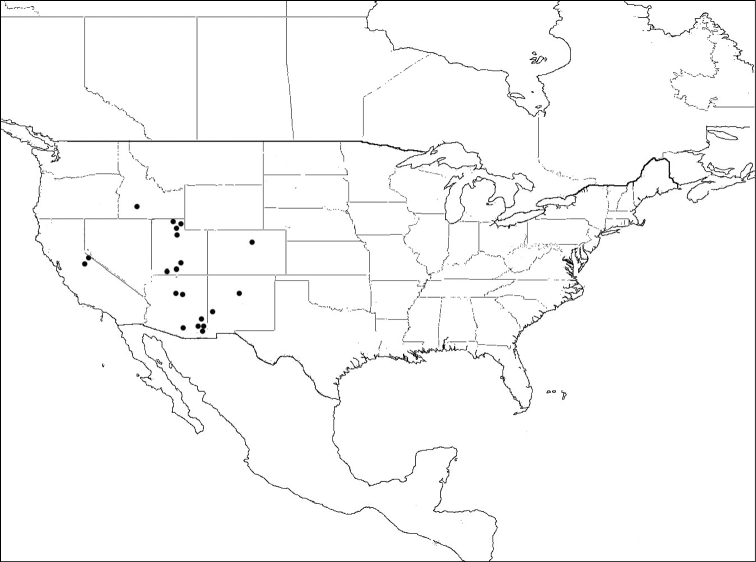
Distribution of *Megachile snowi*.

This species was originally described as a subspecies of *Megachile mendica* (Mitchell, 1935). It is raised to species level herein, based on reliable morphological characters distinguishing it from *Megachile mendica*, and an overlapping range with the latter (Figures 12, 16). Mitchell (1935) found a male *Megachile cleomis* cotype to be misidentified, and previously synonymized it under *Megachile mendica snowi*.See *Megachile texana* comments. *Megachile snowi* is a southwestern North America species (Figure 16).

### Megachile (Litomegachile) texana

Cresson, 1878

http://species-id.net/wiki/Megachile_texana

Megachile texana Cresson, 1878: 125. Holotype male, USA: Texas (ANSP).
Megachile generosa Cresson, 1878: 125. Holotype female, USA: Georgia (ANSP).
Megachile cleomis Cockerell, 1900: 13. Lectotype female (here designated), “USA: NM, E. Las Vegas, July 15 ‘99 Collector: A. Garlick, on Cleome” (UCMC).
Megachile pruinosa Friese, 1903: 246. Syntypes male female, (Repository?). Nec. Perez 1897.
Megachile vernonensis Cockerell, 1912: 354. Holotype male, CANADA: British Columbia (Repository?).

#### Diagnosis.

*Megachile texana* is most similar to *Megachile lippiae* in size and appearance. The chief differences are pubescence coloration and some structural differences in the transverse carina on T6 of the male. *Megachile texana* females have more black setae and pubescence apparent laterally on T2-T6 than *Megachile lippiae* which only has black setae on T4-T6. *Megachile texana* males also have black pubescence on the mesonotum and T2, while *Megachile lippiae* has only white pubescence. Both *Megachile lippiae* and *Megachile texana* have a transverse carina on T6 with a distinct deep medial notch and jagged projections. These carina projections tend to be shorter in *Megachile texana*, whereas the carina of *Megachile lippiae* often has long “fingerlike” projections. *Megachile texana*,

**Female.** Body length11–14 mm. Mandible 4-toothed, with no angulation between teeth 3 and 4 (Figure 4A). T2-4 with deep transverse basal grooves. T1-5 with apical fringes of white hair covering marginal zone. T1 with black discal pubescence medially, white pubescence laterally. T2-5 with black discal pubescence and setae (Figure 5K). T6 with pale appressed pubescence and erect black setae basally. T6 deeply and evenly concave in profile and laterally in dorsal view. S1-4 with ivory setae; S5 with ivory setae basally, black setae apically; S6 with black setae (Figure 5I).

**Male.** Body length10–12 mm. Mandible 3-toothed.Ocellocular distance less than ocelloccipital distance (Figure 4C). Head with white pubescence, vertex with black pubescence. Mesosoma with white pubescence, scutum with black pubescence. T5 with complete apical fringe of white hair covering marginal zone. T6 with tomentum; transverse carina with distinct deep medial notch and short jagged projections; true apical margin with submedial teeth closer to lateral teeth than each other (Figure 6B). Genitalia and hidden sterna shown in Figures 7G1-G4.

#### Variability.

Male tergal discal pubescence variable in color. Pubescence of male mesonotum and head can vary, making it occasionally challenging to differentiate this species from *Megachile lippiae*. Primarily, if there is any black pubescence on the mesonotum, it is *Megachile texana*. If there are no black hairs in this area, it is *Megachile lippiae*. The females of these two species are also sometimes difficult to separate. *Megachile lippiae* can occasionally have black setae laterally on T4 in addition to T5, but if the black setae are present on T3 or T2, then it is *Megachile texana*. *Megachile texana cleomis* was distinguished by the presence of black setae on T3, but that form is now in synonymy under *Megachile texana*.

#### Distribution of material examined.

USA: Arizona: Cochise, Gila and Maricopa Counties (May-Aug.); California: Mariposa, Riverside, Tuolumne and Trinity Counties (Apr.-Jul.); Florida: Alachua, Putnam and Duval Counties (Jun.-Oct.); Mississippi: Oktibbeha County (May); New Mexico: Eddy County (Aug.); New York: New York County (Jun.); Nevada: Clarke, Lincoln and Washoe Counties (Jun.); South Carolina: Chesterfield and Dorchester Counties (May); Texas: Brewster County (Apr.); Utah: Cache, Garfield, Tooele and Washington Counties (Jun.-Sep.); MEXICO: Puebla. 46 females, 57 males.

#### Ecology.

*Megachile texana* utilizes existing nesting sites in the ground and under rocks (Krombein 1970). Observations by Eickwort et al. (1981) showed that these bees also excavate their own nests. The cocoons completely fill their cells and are covered with an outer layer of reddish brown threads and an inner layer of brown threads (Eickwort et al. 1981).

#### Flower records.

*Arctostaphylous patula* (Ericaceae), *Asclepias speciosa* (Asclepiadaceae), *Asclepias syriaca* (Asclepiadaceae), *Baptisia* sp. (Fabaceae)., *Blephilia ciliata* (Lamiaceae), *Calamintha ashei* (Lamiaceae), *Dalea pinnata* (Fabaceae), *Erigeron divergens* (Asteraceae), *Erysimum asperum* (Brassicaceae), *Hemerocallis* sp. (Liliaceae), *Dalea candida* (Fabaceae), *Marrubium vulgare* (Lamiaceae), *Medicago sativa* (Fabaceae), *Melilotus alba* (Fabaceae), *Mentzelia* sp. (Loasaceae), *Opuntia* sp. (Cactaceae), *Phacelia heterophylla* (Hydrophyllaceae), *Phaseolus limensis* (Fabaceae), *Ptelea trifoliata* (Rutaceae), *Ratibida columnaris* (Asteraceae), *Rhus glabra* (Anacardiaceae), *Streptanthus* sp. (Brassicaceae), *Tephrosia virginiana* (Fabaceae), *Trifolium hybridum* (Fabaceae), *Viguiera stenoloba* (Asteraceae), *Vitex agnus castus* (Verbenaceae).

#### Comments.

**Figure 17.**
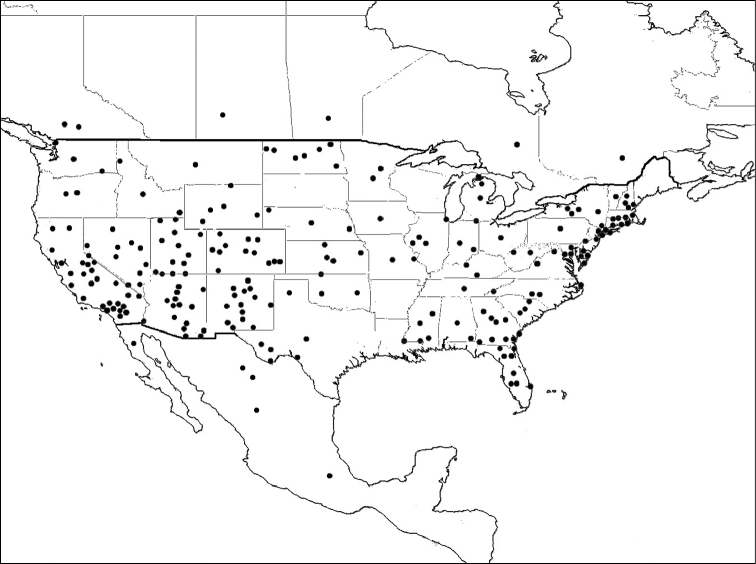
Distribution of *Megachile texana*.

*Megachile cleomis* is one of the synonyms of *Megachile texana*. It was originally described by Cockerell in 1900, based on two cotypes from a locality in New Mexico, a male and a female. The male was later found to be a male *Megachile snowi*. The female is herein designated as the lectotype for *Megachile cleomis*, which remains in synonymy with *Megachile texana*. This situation illustrates the importance of correctly assigning holotypes. *Megachile texana* is a widespread species which is found across North America (Figure 17).

## Conclusions and future directions

There is more work to be done with *Litomegachile*. There are issues regarding types that need to be resolved. Locating types is made easier through the databasing of collections, and there is still more to be done. Repositories for *Megachile palmarum*, *Megachile pruinosa*, and *Megachile vernonensis* are unknown. Neotypes were not designated for *Megachile brevis*, which appears to be missing a holotype, presumed destroyed. The neotype was not designated because of the possibility that it could be in a collection and simply unaccounted for. A lectotype was designated by Cresson in 1916 for *Megachile mendica* but it was not located and so was not examined. Distribution maps and locality data can be greatly refined and expanded. The maps provided here only represent a portion of available collection data. As material from more collections are reliably identified and databased, records that are accurate and available to researchers will greatly improve this field of study. Knowledge of the nesting behavior, ecology, and plant associations of this group remains incomplete. Again, acquisition of additional data will aid compilation of host plant records and more detailed analysis of plant relationships. Additional collecting trips and review and identification of specimens in collections may reveal more diversity. *Megachile pankus* was uncovered in current collections. The male of *Megachile pankus* is unknown, and it is likely that there are more species to be discovered in tropical southern ranges of this group. A phylogeny using molecular and morphological data would further clarify the relationships between the species of this group.

## Supplementary Material

XML Treatment for Megachile (Litomegachile) brevis

XML Treatment for Megachile (Litomegachile) coquilletti

XML Treatment for Megachile (Litomegachile) gentilis

XML Treatment for Megachile (Litomegachile) lippiae

XML Treatment for Megachile (Litomegachile) mendica

XML Treatment for Megachile (Litomegachile) onobrychidis

XML Treatment for Megachile (Litomegachile) pankus

XML Treatment for Megachile pseudobrevis

XML Treatment for Megachile (Litomegachile) snowi

XML Treatment for Megachile (Litomegachile) texana
